# Autistic Traits and Suicidal Thoughts, Plans, and Self-Harm in Late Adolescence: Population-Based Cohort Study

**DOI:** 10.1016/j.jaac.2018.01.023

**Published:** 2018-05

**Authors:** Iryna Culpin, Becky Mars, Rebecca M. Pearson, Jean Golding, Jon Heron, Isidora Bubak, Peter Carpenter, Cecilia Magnusson, David Gunnell, Dheeraj Rai

**Affiliations:** a University of Bristol, Bristol, UK; b Karolinska Institutet, Stockholm, Sweden; c Avon and Wiltshire Partnership National Health Service (NHS) Trust, Bristol, UK

**Keywords:** autism spectrum disorder, suicidal ideation, suicidal behavior, depression, population-based study

## Abstract

**Objective:**

To examine the hypothesis that autism spectrum disorders (ASD) diagnosis and traits in childhood are associated with suicidal thoughts, plans and self-harm at 16 years, and that any observed associations are explained by depression at 12 years.

**Method:**

We examined associations between ASD diagnosis and 4 dichotomized ASD traits (social communication, pragmatic language, repetitive behavior, and sociability) with suicidal and nonsuicidal self-harm, suicidal thoughts, and suicidal plans at age 16 years in 5,031 members of the United Kingdom−based birth cohort study the Avon Longitudinal Study of Parents and Children. We assessed whether any associations were explained by depressive symptoms in early adolescence measured by the Short Moods and Feelings Questionnaire at 12 years.

**Results:**

Children with impaired social communication had a higher risk of self-harm with suicidal intent (relative risk [RR] = 2.14, 95% CI = 1.28–3.58), suicidal thoughts (RR = 1.42, 95% CI = 1.06–1.91), and suicidal plans (RR = 1.95, 95% CI = 1.09–3.47) by age 16 years as compared to those without. There was no evidence for an association between ASD diagnosis and outcomes, although these analyses were imprecise because of small numbers. There was also no evidence of an association between other autism traits and the outcomes. Approximately 32% of the total estimated association between social communication impairment and self-harm was explained by depressive symptoms at 12 years.

**Conclusion:**

Social communication impairments are an important autistic trait in relation to suicidality. Early identification and management of depression may be a preventative mechanism, and future research identifying other potentially modifiable mechanisms may lead to interventions against suicidal behavior in this high-risk group.

Autism spectrum disorders (ASD) are developmental disorders characterized by deficits in social interaction and communication, restricted range of interests, and repetitive behaviors.^1^ An increase in premature mortality in this population has been recently reported, with suicide being suggested as a significant contributor.^2^ However, there is a lack of population-based research on suicidal behavior and suicidal ideation in this population.^3^ Suicidal behavior describes self-harm (with or without suicidal intent) and completed suicide, whereas suicidal ideation refers to suicidal thoughts and cognitions.^3^ Self-harm and suicidal behavior are highly prevalent in young people,^4^ and self-harming behaviors are a strong risk factor for completed suicide.^5^

The possibility of higher rates of suicidal ideation and attempts in individuals with ASD has been reported^6^, ^7^; however, the existing research has focused mostly on either case reports^8^ or cross-sectional studies carried out in clinical^7^, ^9^ and nonclinical^10^ settings. The cross-sectional design,^7^, ^9^, ^10^ selective nature of the samples,^7^ and lack of an adequate comparison group^9^ increase the likelihood of selection bias and limit the generalizability of these findings. There remains a lack of longitudinal research using large population-based samples while accounting for possible confounding factors and reducing the possibility of selection and recall bias. It is also important to distinguish between self-harm with and without suicidal intent, as these, although related, are clinically distinct outcomes.^11^ In addition, a growing body of research argues that the social, communication, and behavioral difficulties comprising the autism spectrum may have distinct etiologies,^12^ and it is plausible that outcomes related to difficulties in individual autistic traits may also differ. To our knowledge, there have been no prospective cohort studies examining the association between autistic traits and suicidal behavior and ideation.

Furthermore, the mechanisms underlying any associations between autism/autistic traits and suicidality have not been examined. For example, depression is a strong risk factor for suicidal ideation and self-harm in the general population^13^; however, whether it could explain a greater risk of suicidal thoughts or behaviors in people with autism has not been studied. Quantifying this relationship is important, as it may inform preventive or intervention strategies, considering that depression is potentially treatable. We used prospectively collected data from the Avon Longitudinal Study of Parents and Children (ALSPAC), a large birth cohort in Bristol, UK, to address some of these gaps in the literature. Our research questions were: as follows: (1) Is an autism diagnosis and/or are autistic traits associated with suicidal ideation (suicidal thoughts and plans) and suicidal behavior (self-harm with and without suicidal intent) by age 16 years? (2) Are any of the observed associations explained by depressive symptoms in early adolescence?

## Method

### Participants

The sample comprised participants from the Avon Longitudinal Study of Parents and Children (ALSPAC). During phase I enrollment, a total of 14,541 pregnant mothers residing in the former Avon Health Authority in the southwestern part of England with expected dates of delivery between April 1, 1991, and December 31, 1992, were recruited. These pregnancies resulted in 14,062 live births, and 13,988 children were alive at 1 year of age. When the oldest children were approximately 7 years of age, an attempt was made to bolster the initial sample with eligible case individuals who had failed to join the study originally. The total sample size for analyses using data after the age of 7 years is 15,247 pregnancies, of which 14,701 children were alive at 1 year of age. Ethical approval for the data collection was obtained from the ALSPAC Ethics and Law Committee and the Local Research Ethics Committees. Detailed information about the cohort has been collected since early pregnancy, including regular self-completion questionnaires from mothers and children. Information about ALSPAC is available at www.bristol.ac.uk/alspac/, including a searchable data dictionary (http://www.bris.ac.uk/alspac/researchers/data-access/data-dictionary/). Further details on the cohort profile, representativeness, and phases of recruitment are provided by Boyd *et al.*^14^ and Fraser *et al.*^15^

### Measures

#### ASD Diagnosis and Autistic Traits

Identification of children diagnosed with ASD in ALSPAC has been described in detail elsewhere.^16^, ^17^ Briefly, a multisource approach included a record linkage study identifying cases from the following: community pediatric records; autism as the main reason for special educational needs from school records; maternal reports at age 9 years that the child had been diagnosed with “an autistic spectrum disorder or Asperger syndrome”; free text questionnaire responses from 6 months to 11 years; and ad hoc letters from parents to the Study Director.^16^ The diagnosis of ASD in ALSPAC has been previously validated by a consultant pediatrician using the International Classification of Diseases (ICD-10),^16^ and we have cross validated cases ascertained from maternal reports against autistic spectrum traits.^17^

Four individual measures optimally predictive of autism diagnosis in ALSPAC^18^ via parental questionnaires were analyzed. These included the following: the Social and Communication Disorder Checklist (assessed at 91 months); a measure of repetitive behavior (assessed at 69 months); the sociability subscale of the Emotionality, Activity and Sociability Temperament Scale (assessed at 38 months); and the coherence subscale of the Children’s Communication Checklist (assessed at 115 months) (see Steer *et al.*^18^ for description of measures). Consistent with previous research using the ALSPAC data, each ASD trait was dichotomized to create the high-risk group (for ASD) of as close as possible to 10% of the population.^17^

#### Self-Harm and Suicidal Thoughts and Plans

Self-harm questions were based on those used in the Child and Adolescent Self-Harm in Europe Study (CASE).^4^ Participants who responded positively to the question “Have you ever hurt yourself on purpose in any way (e.g., by taking an overdose of pills or by cutting yourself?)” at 16 years were classified as having a lifetime history of self-harm. Responses to 2 additional questions were used to identify those who self-harmed with suicidal intent: (1) selecting the response option “I wanted to die” in response to the question “Do any of the following reasons help to explain why you hurt yourself on that (i.e., the most recent), occasion?” or (2) a positive response to the question “On any of the occasions when you have hurt yourself on purpose, have you ever seriously wanted to kill yourself?” These questions helped to identify individuals who had harmed with suicidal intent at some point during their lifetime, and those who had only ever engaged in nonsuicidal self-harm. Self-harm behaviors were classified according to individual’s self-reported suicidal intent.^19^

Lifetime history of suicidal thoughts and plans were also assessed with the following questions at 16 years: “Have you ever thought of killing yourself, even if you would not really do it?” and “Have you ever made plans to kill yourself?”

#### Mediating Variable

To examine whether depressive symptoms in early adolescence mediate the association between childhood ASD and suicidal behavior in late adolescence, we used data from the Short Mood and Feelings Questionnaire (SMFQ), a 13-item instrument used to evaluate core depressive symptomatology in children and adolescents 8 to 18 years of age,^20^ assessed at 12 years. The SMFQ correlates highly with more extensive depression diagnostic tool such the Diagnostic Interview Schedule for Children.^21^

#### Confounding Variables

Parental, socioeconomic, and family characteristics identified in previous studies as being associated with ASD and suicidal behavior were collected prospectively from maternal questionnaires during the antenatal period. These included the following: financial problems (occurrence of major financial problems since pregnancy versus none); highest maternal educational attainment (minimal education or none, compulsory secondary level, up to age 16 years); noncompulsory secondary level (up to age 18 years) versus university level education); parental social class (professional/managerial versus manual), with the highest of maternal or paternal social class used; and accommodation type (detached house or semidetached house versus a flat/apartment); maternal age (in years); maternal antenatal (18 and 32 weeks’ gestation) and early postnatal (8 weeks and 8 months) depression assessed using the Edinburgh Postnatal Depression Scale (EPDS); maternal antenatal anxiety (18 and 32 weeks’ gestation) measured using anxiety items from the Crown-Crisp Index, a validated self-rating inventory; parental suicide attempt assessed using maternal questionnaires repeated 8 times from birth to 11 years (yes or no); sexual abuse (repeated 7 times from birth to 8 years) and physical cruelty (repeated 8 times from birth to 11 years) to children in the household by mother/partner (yes or no). Analyses were also adjusted for child’s sex (male versus female) and ethnicity (white versus nonwhite).

### Statistical Analyses

#### Main Effects

First, we compared characteristics of children in the study with missing data with those who comprised the study sample and carried out descriptive analysis. In the main analysis, we used multinomial regression to examine associations with explanatory variables and a 3-category self-harm outcome: no self-harm; self-harm without suicidal intent; and self-harm with suicidal intent at 16 years. We used modified Poisson regression to examine the associations between explanatory variables and suicidal thoughts and plans at 16 years as binary outcomes to derive relative risks and robust 95% confidence intervals. A modified Poisson regression approach (with a robust error variance) directly estimates relative risks and robust error estimates with binary outcomes.^22^ We tested models unadjusted and adjusted for the potential confounding factors. In accordance with ALSPAC policy to protect confidentiality, analyses in which the number of participants was less than 5 in a given cell were censored. All analyses were conducted using Stata v.13 (StataCorp, College Station, TX).

#### Mediating Effects

We wanted to assess the importance of depressive symptoms at age 12 years in explaining the association between autistic traits in childhood and self-harm at age 16 years. Direct pathways (ASD to risk of self-harm) and indirect pathways (through depressive symptoms) were estimated using structural equation modeling in Mplus v.7. Confirmatory factor analysis was used to derive a normally distributed latent trait underlying the observed SMFQ^20^ scores using ordinal response items. A latent trait approach helps account for measurement error and increases power by modeling variables as a continuous trait.^23^ The approach recommended by Muthén (unpublished manuscript, October 28, 2011; available online https://pdfs.semanticscholar.org/776d/2df784e67ab691bbffc280d3f4a480740300.pdf) was used to estimate mediation effects within the context of possible confounding. Analyses were adjusted for a range of individual, maternal, and familial confounders. A detailed description of the mediation method is presented in Supplement 1, available online.

#### Missing Data

Similar to all longitudinal birth cohort studies with long-term follow-up, attrition is a well-known problem in ALSPAC. Thus, all missing data were imputed, and all analyses were repeated using data for n = 5,093 (main effects) and n = 7,788 (mediation) adolescents. We imputed for missing data because ignoring those cases with missing data can result in bias by making the assumption that data are missing completely at random.^24^ A complete description of the imputation method is presented in Supplement 2, available online.

## Results

### Sample Derivation

**Figure 1:**
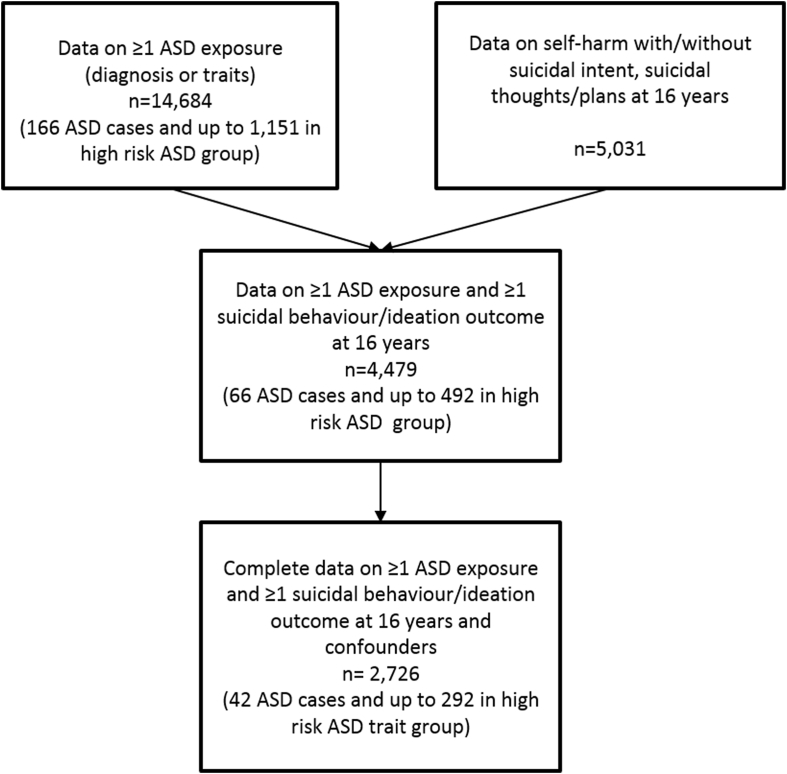
Study Sample ***Note:****ASD = autism spectrum disorder.*

Our starting sample included those individuals with data on at least one ASD exposure (presence/absence of ASD diagnosis or data on at least one of the 4 dichotomized ASD traits; n = 14,684). The number of adolescents with complete data on depressive symptoms at age 12 years was 6,680. Complete outcome data on suicidal behavior and ideation at age 16 years were available for 5,031 adolescents. The numbers of participants available with data on suicidal behavior and ideation at 16 years, childhood ASD exposures, and confounders are shown in Figure 1. Descriptive statistics of the sample by the presence of autism/autistic traits are presented in Table S1, available online. Characteristics of the sample by the completeness of data availability are presented in Table S2, available online.

### Main Effects

Of 5,031 adolescents with data on suicidal behavior and ideation up to age 16 years, 602 (11.9%, 95% CI = 11.0–12.8%) reported self-harm without suicidal intent, 347 (6.9%, 95% CI = 6.2%–7.6%) reported self-harming with suicidal intent, 797 (15.7%, 95% CI = 14.6–16.4) reported experiencing suicidal thoughts, and 227 (4.5%, 95% CI = 3.8–5.0) reported making suicidal plans (Tables 1 and 2). The regression analysis (Table 3) provided evidence for an effect of impaired social communication on risk of self-harm with suicidal intent (adjusted relative risk [RR] = 2.14, 95% CI = 1.28–3.58, *p* = .004), but not self-harm without suicidal intent (adjusted RR = 1.02, 95% CI = 0.62–1.67, *p* = .943). There was also evidence for an effect of impaired social communication on risk of suicidal thoughts (adjusted RR = 1.42, 95% CI = 1.06–1.91, *p* = .019) and suicidal plans (adjusted RR = 1.95, 95% CI = 1.09–3.47, *p* = .024) (Table 4). There was no evidence of an association between ASD diagnosis and any of the outcomes, although the numbers were very low and confidence intervals wide. None of the other autistic traits (sociability, coherence, and repetitive behavior) appeared to be associated with the outcomes (Tables 3 and 4).

**Table 1:** Prevalence of Self-Harm With and Without Suicidal Intent at 16 Years in Young Adults by Autism Spectrum Disorder (ASD) and Autistic Trait Measures

Exposure/Risk Group (Age of Assessment)	No Self-Harm (n = 4,114)	Self-Harm Without Suicidal Intent (n = 602)	Self-Harm With Suicidal Intent (n = 347)	*p*
ASD diagnosis (diagnosed by 11 y), n (%)				
No ASD diagnosis	4,054 (81.2%)	595 (11.9%)	343 (6.9%)	—
ASD diagnosis	60 (84.5%)	Censored	Censored	
Reduced social communication (91 mo), n (%)				
Lower risk	3,220 (82.3%)	460 (11.8%)	233 (5.9%)	<.001
Higher risk	249 (76.2%)	37 (11.3%)	41 (12.5%)	
Repetitive behavior (69 mo), n (%)				
Lower risk	3,254 (81.6%)	477 (11.9%)	258 (6.5%)	.074
Higher risk	212 (80.3%)	26 (9.8%)	26 (9.9%)	
Reduced sociability (38 mo), n (%)				
Lower risk	3,257 (81.7%)	463 (11.6%)	269 (6.7%)	.833
Higher risk	408 (82.8%)	54 (10.9%)	31 (6.3%)	
Reduced coherence (115 mo), n (%)				
Lower risk	3,311 (81.7%)	491 (12.1%)	250 (6.2%)	<.001
Higher risk	302 (79.7%)	34 (9.0%)	43 (11.3%)	

Note: *p* Values based on χ^2^ test of the association between self-harm with/without suicidal intent and categorical ASD exposures.

Censored to prevent disclosure because of small cell counts.

**Table 2:** Prevalence of Suicidal Thoughts and Plans at 16 Years in Young Adults With Autism Spectrum Disorder (ASD) and Autistic Trait Measures

Exposure/Risk group (Age of Assessment)	Suicidal Thoughts		*p*	Suicidal Plans		*p*
	No (n = 4,269)	Yes (n = 797)		No (n = 4,840)	Yes (n = 227)	
ASD diagnosis (diagnosed by 11 y), n (%)						
No ASD diagnosis	4,207 (84.2%)	787 (15.8%)	.665	4,769 (95.5%)	226 (4.5%)	—
ASD diagnosis	62 (86.1%)	10 (13.9%)		Censored	Censored	
Reduced social communication (91 mo), n (%)						
Lower risk	3,351 (85.5%)	569 (14.5%)	<.001	3,773 (96.2%)	149 (3.8%)	.002
Higher risk	258 (78.9%)	69 (21.1%)		302 (92.6%)	24 (7.4%)	
Repetitive behavior (69 mo), n (%)						
Lower risk	3,387 (84.9%)	603 (15.1%)	.198	3,829 (95.9%)	163 (4.3%)	.070
Higher risk	218 (81.9%)	48 (18.1%)		249 (93.6%)	17 (6.4%)	
Reduced sociability (38 mo), n (%)						
Lower risk	3,384 (84.7%)	611 (15.3%)	.759	3,825 (95.7%)	173 (4.3%)	.360
Higher risk	415 (84.2%)	78 (15.8%)		476 (96.5%)	17 (3.5%)	
Reduced coherence (115 mo), n (%)						
Lower risk	3,432 (84.7%)	619 (15.3%)	.705	3,891 (96.0%)	161 (4.0%)	.016
Higher risk	320 (84.0%)	61 (16.0%)		356 (93.4%)	25 (6.6%)	

Note: *p* Values based on χ^2^ test of the association between suicidal thoughts, plans, and categorical ASD exposures.

Censored to prevent disclosure because of small cell counts.

**Table 3:** Associations Between Autism Spectrum Disorder (ASD Versus No ASD), Autistic Trait Measures (High Versus Low ASD Risk Group), and Self-Harm With/Without Suicidal Intent^a^ (Versus No Self-Harm) at 16 Years

Exposure/Risk Group (Age of Assessment)	Total n^b^	Unadjusted						Adjusted^c^					
		Self-Harm Without Suicidal Intent			Self-Harm With Suicidal Intent			Self-Harm Without Suicidal Intent			Self-Harm With Suicidal Intent		
		RR	(95% CI)	*p*	RR	(95% CI)	*p*	RR	(95% CI)	*p*	RR	(95% CI)	*p*
ASD diagnosis (diagnosed by 11 y)	2,720	0.34	(0.08, 1.43)	.142	Too few observations			0.37	(0.09, 1.56)	.175	Too few observations		
Reduced social communication (91 mo)	2,651	0.89	(0.55, 1.43)	.622	2.10	(1.28, 3.34)	.003	1.02	(0.62, 1.67)	.943	2.14	(1.28, 3.58)	.004
Repetitive behavior (69 mo)	2,675	0.63	(0.35, 1.16)	.138	1.40	(0.77, 2.53)	.268	0.68	(0.37, 1.26)	.222	1.31	(0.69, 2.46)	.407
Reduced sociability (38 mo)	2,696	0.88	(0.59, 1.30)	.512	0.95	(0.56, 1.59)	.840	0.77	(0.45, 1.29)	.318	1.60	(0.94, 2.74)	.084
Reduced coherence (115 mo)	2,669	0.65	(0.39, 1.09)	.102	1.51	(0.91, 2.49)	.110	0.94	(0.63, 1.41)	.773	1.02	(0.60, 1.75)	.935

Note: RR = relative risk.

a Suicidal self-harm refers to lifetime self-harm with suicidal intent; individuals in this group may also have engaged in episodes of nonsuicidal self-harm.

b Analyses restricted to individuals with complete data on exposure, outcome and confounders.

c Adjusted for child characteristics (sex and ethnicity), socioeconomic position (financial problems, maternal educational attainment, parental social class, and accommodation type), maternal characteristics and mental health (maternal age, antenatal anxiety, and antenatal and early postnatal depression), family history of suicide (parental suicide attempt); and early adverse experiences (childhood sexual abuse and physical cruelty to children in the household by mother/partner).

**Table 4:** Associations Between Autism Spectrum Disorder (ASD Versus No ASD), Autistic Trait Measures (High Versus Low ASD Risk Group), and Suicidal Thoughts (Versus No Suicidal Thoughts) and Plans (Versus No Suicidal Plans) at 16 Years

Exposure/Risk group (Age of Assessment)	Total n^a^	Unadjusted			Adjusted^b^			Total n^a^	Unadjusted			Adjusted^b^		
		Suicidal Thoughts							Suicidal Plans					
		RR	(95% CI)	*p*	RR	(95% CI)	*p*		RR	(95% CI)	*p*	RR	(95% CI)	*p*
ASD diagnosis (diagnosed by 11 y)	2,726	0.32	(0.08, 1.25)	.101	0.43	(0.11, 1.73)	.238	2,728	Too few observations			Too few observations		
Reduced social communication (91 mo)	2,658	1.41	(1.04, 1.90)	.024	1.42	(1.06, 1.91)	.019	2,659	1.89	(1.07, 3.33)	.027	1.95	(1.09, 3.47)	.024
Repetitive behavior (69 mo)	2,681	1.23	(0.86, 1.76)	.250	1.20	(0.85, 1.69)	.299	2,683	1.41	(0.70, 2.87)	.331	1.44	(0.75, 2.74)	.270
Reduced sociability (38 mo)	2,702	1.10	(0.83, 1.47)	.493	1.16	(0.87, 1.55)	.305	2,704	0.83	(0.42, 1.64)	.601	0.87	(0.44, 1.72)	.689
Reduced coherence (115 mo)	2,674	0.84	(0.58, 1.22)	.370	0.90	(0.63, 1.29)	.577	2,676	1.00	(0.49, 2.03)	1.00	1.00	(0.49, 2.02)	.979

Note: RR = relative risk.

a Analyses restricted to individuals with complete data on exposure, outcome, and confounders.

b Adjusted for child characteristics (sex and ethnicity), socioeconomic position (financial problems, maternal educational attainment, parental social class, and accommodation type), maternal characteristics and mental health (maternal age, antenatal anxiety, and antenatal and early postnatal depression), family history of suicide (parental suicide attempt), and early adverse experiences (childhood sexual abuse and physical cruelty to children in the household by mother/partner).

Results were comparable when using imputed data sets for the main effect of impaired social communication on the outcomes (see Table S3, Table S4, Table S5, available online). In these imputed analyses, there was evidence for the main effect of repetitive behavior and reduced coherence on risk of self-harm with suicidal intent (relative risk [RR] = 1.58, 95% CI = 1.00–2.48, *p* = .049, and RR = 1.97, 95% CI = 1.36–2.81, *p* < .001 respectively), and suicidal plans (RR = 1.63, 95% CI = 1.03–2.58, *p* = .035, and RR = 1.61, 95% CI = 1.08–2.41, *p* = .019 respectively).

### Mediation Effects

**Figure 2:**
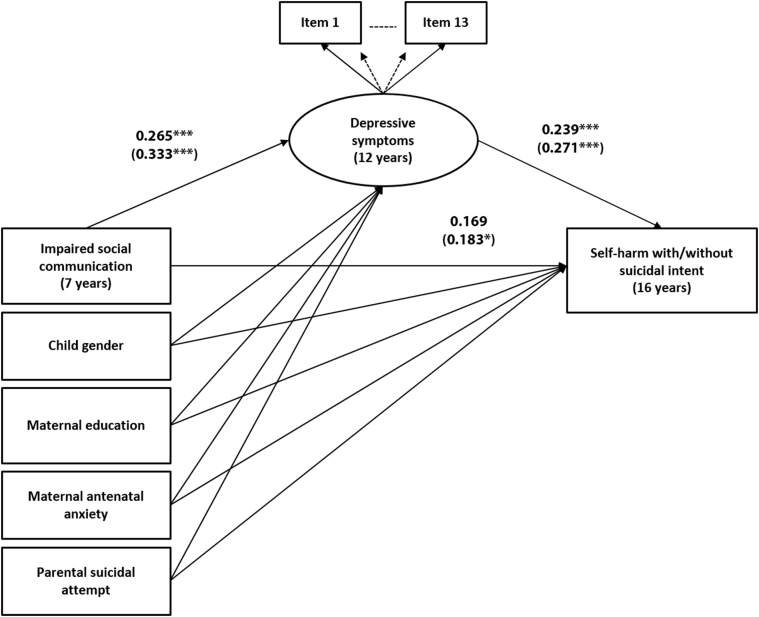
Structural Mediation Model Estimating the Direct Effect of Impaired Social Cognition on Lifetime Self-Harm at 16 Years and the Indirect Effect Through Child’s Depressive Symptoms at 12 Years (Adjusted for Potential Child, Maternal, and Socioeconomic Confounders) ***Note:***Path estimates on the edges are adjusted unstandardized regression coefficients (β). Observed variables are represented by squares, whereas the latent variable is represented by a circle. Covariances are not shown to reduce figure complexity. Paths coefficients in brackets are from the imputed data analysis. *p ≤ .05; ***p *< .001.*

Given the main effect of impaired social communication on risk of self-harm, we examined whether depressive symptoms in early adolescence mediate this association. To estimate the mediation model, we combined suicidal/nonsuicidal self-harm into one category. First, we examined the fit of the measurement model incorporating exposure, mediator, and confounders. The full model was run without using bootstrapping to enable calculation of model fit statistics. The root mean square error of approximation (RMSEA) of 0.04 (95% CI = 0.03–0.04) and the comparative fit index (CFI) of 0.97 indicated that the measurement model fit the data well, supporting the adequacy of the model for tests of structural paths and mediation effects (Figure 2). We estimated unadjusted and adjusted structural mediation models to examine the direct and indirect effects of impaired social cognition on self-harm through depressive symptoms while accounting for possible mediator−outcome confounding. Mediation models were adjusted for child (sex), socioeconomic (maternal educational attainment), maternal (antenatal anxiety), and familial (parental suicidal attempt) characteristics that had statistical evidence of association with exposure and outcome in the main effects analyses. There was evidence of an indirect pathway from impaired social cognition to self-harm via depressive symptoms (unadjusted product coefficient [β] = 0.087, 95% CI = 0.03–0.14, *p* = .002). There was no evidence of a direct pathway from impaired social cognition to self-harm once the indirect effect via depressive symptoms was accounted for (unadjusted regression coefficient [β] = 0.090, 95% CI = −0.11 to 0.28, *p* = .372). This indirect pathway via depressive symptoms accounted for 32% of the total estimated association between impaired social cognition and self-harm. Adjustment for the confounders made little difference to the parameter estimates (Table 5).

**Table 5:** Estimates of the Direct Effect and Effect Mediated Through Depressive Symptoms in the Association Between Impaired Social Cognition and Self-Harm at 16 Years, Unadjusted and Adjusted for Antenatal Confounders and Child’s Sex (n = 2,936)^a^

Effect Size^b^	Model Estimates					
	Unadjusted Model			Adjusted Model^c^		
	*β*	(95% CI)	*p*	*β*	(95% CI)	*p*
Total Effect Reduced social communication on self-harm	0.177	(−0.02 to 0.38)	.084	0.250	(0.05 to 0.45)	.016
Indirect Effect Reduced social communication on self-harm, through depressive symptoms	0.087	(0.03 to 0.14)	.002	0.081	(0.03 to 0.13)	.001
Direct Effect Reduced social communication on self-harm, adjusted for depressive symptoms	0.090	(−0.11 to 0.28)	.372	0.169	(−0.03 to 0.37)	.102

Note:

a Analyses restricted to participants with complete exposure, outcome, mediator, and confounders.

b Effect size are unadjusted and adjusted regression coefficients (β unstandardized).

c Adjusted for child (child’s sex), socioeconomic (maternal educational attainment), maternal (antenatal anxiety), and familial (parental suicidal attempt) characteristics.

The direct and indirect estimates with imputed data sets led to similar results (see Table S5, available online). However, the sizes of the observed direct and indirect effects were greater, which may suggest that attrition led to an underestimation of the direct and indirect effect sizes in the complete case analyses. Based on the imputed analyses, a slightly higher proportion (35%) of the total association between impaired social cognition and self-harm was accounted for by the indirect path through depressive symptoms (see Supplement 3, available online).

## Discussion

To our knowledge, this is the first large population-based study to investigate the association between an autism diagnosis and traits and suicidal ideation and behavior by late adolescence, as well as examining the mechanisms of this association. We did not find an association with diagnosed autism and the outcomes, although the estimates were imprecise because of small numbers. Impairments in social communication were associated with an increased risk of suicidal thoughts, suicidal plans, and self-harm with suicidal intent, but not self-harm without suicidal intent.

Our findings suggest that social communication difficulties may be important in relation to suicidality. Our findings are consistent with existing case studies suggesting that social impairments and difficulties in establishing interpersonal relationships are triggers for suicidal behavior.^25^ Suicidal behavior in individuals with autism is often underreported,^26^ particularly in those with impaired communicative abilities and comorbid self-injurious behavior.^27^ Our findings emphasize the potential importance of assessing whether self-injurious behavior occurs in the context of suicidal ideation.^3^ The stronger associations for social communication with suicidality outcomes as compared to the other traits of autism is concordant with the argument on the fractionation of core autistic impairments.^12^ However, it is important to note that although difficulties in social communication are a key impairment in autism, they could also be observed in other conditions, and may be a problem in their own right. Thus, impairments in social communication may also be considered a trait associated with higher suicidality, inside or outside of the context of ASD.

It has been argued that it is adolescents with ASD without intellectual disability who are at most risk of suicidal ideation and behaviour,^28^ because of the increased awareness of their social difficulties and secondary depression associated with social isolation and exclusion.^7^ We were not able to directly test this possibility, as participants in our sample were predominantly high-functioning individuals (96.4% of individuals with autism diagnosis had an IQ of >70), and we did not have enough statistical power to study lower- and higher-functioning individuals separately.

We tested whether depression during early adolescence could explain the association between social communication and suicidal behavior. Children with impaired social communication skills were at increased risk for depressive symptoms in early adolescence, which, in turn, was a strong risk factor for suicidal behavior later in adolescence. It should be noted that although depression explained about a third of the variance of the association between childhood autistic traits and suicidal behavior, substantial variance remained unexplained. This finding emphasises the need for identifying other individual and environmental, potentially modifiable mechanisms (e.g., anxiety, peer victimization/bullying) in this relationship.

The strengths of this study include the large sample, the long-term follow-up, the availability of data on several outcomes, as well as rich data on confounders, and a longitudinal design that permits an examination of mediating pathways. Furthermore, we were able to examine a range of autistic traits in a large population,^3^ despite a relatively small number of cases with an ASD diagnosis. The findings need to be interpreted in light of several limitations. First, our study was likely to be underpowered to detect the association between ASD diagnosis and the outcomes because of a relatively small number of diagnosed cases followed up until age 16 years. Second, despite the population-based sample, it is not possible to rule out selection bias in relation to baseline recruitment or attrition in the sample over time. We attempted to address this by controlling for factors known to be predictive of attrition in ALSPAC and by imputing missing data. The pattern of missing data and imputed analyses suggests that attrition may have led to an underestimation of the size of the association between ASD traits, in particular repetitive behavior and impaired speech coherence, and suicidal ideation and behavior. Third, there are limitations in establishing suicidal intent accompanying self-harm, particularly using self-reports, which could be influenced by fluctuations in mood or change over time. This could be further compounded in individuals with autistic features, who may experience additional difficulties understanding or responding to such questionnaires. These difficulties in expressing emotions and possible lack of verbal skills to communicate thoughts may also make the diagnosis of suicidal ideation problematic, necessitating adjustments to existing clinical diagnostic tools and therapeutic approaches.^29^

In summary, this study suggests that children with impairments in social communication are at higher risk for suicidal ideation and behavior in late adolescence. Depressive symptoms in early adolescence partially explain this association, which emphasizes the importance of addressing the mental health needs of children with autism. Future research is required to assess whether other modifiable mechanisms could be identified, as these may have the potential to lead to preventive action or interventions against suicidal behavior in this high-risk group.

## Figures and Tables

**Table S1:** Associations Between Autism Spectrum Disorder (ASD) and Autistic Trait Measures and Sociodemographic, Parental, and Familial Confounders

Confounders	ASD Diagnosis (Diagnosed by 11 y)		Reduced Social Cognition (91 mo)		Repetitive Behavior (69 mo)		Reduced Sociability (38 mo)		Reduced Coherence (115 mo)	
	No	Yes	No	Yes	No	Yes	No	Yes	No	Yes
Child characteristics										
Sex, n (%)										
Male	7,502 (51%)	133 (80%)	3,652 (50%)	517 (65%)	4,023 (51%)	381 (63%)	4,514 (51%)	668 (58%)	3,561 (49%)	538 (64%)
Female	7,186 (49%)	33 (20%)	3,654 (50%)	281 (35%)	3,936 (49%)	226 (37%)	4,371 (49%)	483 (42%)	3,706 (51%)	298 (36%)
χ^2^, *p* value	55.43, <.001		63.09, <.001		33.72, <.001		21.34, <.001		70.69, <.001	
Ethnicity, n (%)										
White	Censored	Censored	6,704 (96%)	726 (96%)	7,327 (96%)	535 (94%)	8,088 (96%)	1,031 (96%)	6,420 (96%)	727 (97%)
Nonwhite	Censored	Censored	262 (4%)	32 (4%)	286 (4%)	35 (6%)	341 (4%)	43 (4%)	245 (4%)	25 (3%)
χ^2^, *p* value	—		0.39, .529		7.99, .005		0.01, .948		0.24, .626	
Socioeconomic position										
Parental social class, n (%)										
Professional/managerial	5,808 (51%)	97 (67%)	3,843 (57%)	371 (52%)	4,076 (55%)	307 (57%)	4,427 (54%)	505 (49%)	3,684 (57%)	366 (52%)
Manual	5,606 (49%)	47 (33%)	2,931 (43%)	350 (48%)	3,307 (45%)	234 (43%)	3,715 (46%)	529 (51%)	2,800 (43%)	336 (48%)
χ^2^, *p* value	15.45, <.001		7.37, .007		0.48, .487		11.30, .001		5.64, .018	
Accommodation type, n (%)										
Detached house	1,870 (17%)	26 (19%)	1,294 (19%)	124 (17%)	1,372 (18%)	85 (16%)	1,493 (18%)	167 (16%)	1,230 (19%)	132 (18%)
Semi-detached house	7,598 (69%)	105 (75%)	4,831 (71%)	502 (69%)	5,297 (71%)	382 (70%)	5,796 (70%)	759 (71%)	4,648 (71%)	485 (68%)
Flat	1,490 (14%)	8 (6%)	714 (10%)	98 (14%)	814 (11%)	77 (14%)	968 (12%)	141 (13%)	661 (10%)	101 (14%)
χ^2^, *p* value	7.23, .027		7.12, .028		6.98, .031		5.02, .081		10.86, .004	
Maternal educational attainment, n (%)										
Degree	1,583 (14%)	26 (18%)	1,162 (17%)	105 (14%)	1,198 (16%)	89 (16%)	1,256 (15%)	136 (13%)	1,138 (17%)	101 (14%)
A-level	2,760 (24%)	41 (28%)	1,843 (27%)	179 (25%)	1,966 (27%)	140 (25%)	2,140 (26%)	228 (22%)	1,769 (27%)	187 (26%)
O-level	5,496 (47%)	60 (42%)	3,119 (46%)	341 (47%)	3,425 (46%)	249 (44%)	3,853 (47%)	497 (48%)	2,985 (46%)	328 (45%)
Less than O-level	1,733 (15%)	18 (12%)	711 (10%)	105 (14%)	833 (11%)	84 (15%)	957 (12%)	181 (17%)	650 (10%)	110 (15%)
χ^2^, *p* value	4.82, .186		14.03, .003		7.21, .065		34.07, <.001		22.08, <.001	
Financial problems, n (%)										
No financial problems	10,320 (78%)	119 (79%)	5,914 (83%)	594 (76%)	6,429 (82%)	448 (76%)	7,057 (81%)	890 (79%)	5,661 (83%)	599 (77%)
Financial problems	2,888 (22%)	32 (21%)	1,247 (17%)	185 (24%)	1,408 (18%)	143 (24%)	1,646 (19%)	238 (21%)	1,188 (17%)	179 (23%)
χ^2^, *p* value	0.039, .842		19.07, <.001		14.21, <.001		3.08, .079		15.23, <.001	
Early adversity										
Parental history of suicide										
No Parental suicide attempt, n (%)	7,911 (98%)	Censored	6,354 (99%)	665 (97%)	6,883 (98%)	496 (96%)	6,852 (98%)	885 (99%)	6,138 (98%)	663 (97%)
Parental suicide attempt	144 (2%)	Censored	93 (1%)	21 (3%)	111 (2%)	18 (4%)	127 (2%)	10 (1%)	98 (2%)	23 (3%)
χ^2^, *p* value	—		10.33, .001		10.40, .001		2.29, .130		11.42, .001	
Childhood sexual abuse, n (%)										
No Child sexually abused	8,631 (99%)	Censored	6,699 (99%)	711 (99%)	7,523 (99%)	558 (98%)	7,398 (99%)	953 (99%)	6,385 (99%)	702 (99%)
Childhood sexual abuse	48 (1%)	Censored	29 (1%)	6 (1%)	36 (1%)	9 (2%)	44 (1%)	6 (1%)	30 (1%)	6 (1%)
χ^2^, *p* value	—		2.28, .131		11.82, .001		0.02, .896		1.83, .176	
Parental cruelty to children, n (%)										
No	4,799 (95%)	Censored	4,259 (96%)	401 (91%)	4,447 (96%)	290 (90%)	4,272 (95%)	546 (96%)	4,267 (95%)	424 (93%)
Yes	235 (5%)	Censored	183 (4%)	40 (9%)	205 (4%)	32 (10%)	211 (5%)	25 (4%)	201 (5%)	31 (7%)
χ^2^, *p* value	–		22.56, <.001		20.30, <.001		0.12, .726		4.93, .026	
Maternal age and mental health										
Maternal age, mean (SD)	27.3 (5.0)	28.6 (4.8)	28.3 (4.6)	27.9 (4.7)	28.1 (4.6)	28.0 (4.8)	27.9 (4.7)	27.8 (4.6)	28.3 (4.6)	28.1 (4.7)
ANOVA, *p* value	−3.257, 0.001		2.31, 0.021		0.42, 0.677		0.56, 0.577		0.85, 0.394	
Maternal antenatal anxiety, mean (SD)	4.93 (3.6)	5.04 (3.5)	4.58 (3.3)	5.51 (3.7)	4.7 (3.4)	5.7 (3.5)	4.7 (3.5)	4.8 (3.5)	4.6 (3.4)	5.2 (3.7)
ANOVA, *p* value	−0.37, .712		−6.92, <.001		−6.49, <.001		−0.13, .898		−4.70, <.001	
Maternal antenatal depression, n (%)										
No	8,688 (76%)	99 (73%)	5,404 (82%)	486 (69%)	5,816 (81%)	384 (72%)	6,330 (80%)	796 (79%)	5,168 (82%)	516 (74%)
Yes	2,697 (24%)	37 (27%)	1,175 (18%)	222 (31%)	1,395 (19%)	146 (28%)	1,628 (20%)	216 (21%)	1,160 (18%)	182 (26%)
χ^2^, *p* value	0.92, .338		75.14, <.001		20.83, <.001		0.43, .511		24.39, <.001	
Maternal early postnatal depression, n (%)										
No	8,957 (84%)	111 (85%)	5,862 (88%)	537 (74%)	6,346 (87%)	432 (79%)	6,903 (85%)	895 (86%)	5,626 (87%)	552 (78%)
Yes	1,740 (16%)	20 (15%)	826 (12%)	193 (26%)	983 (13%)	114 (21%)	1,183 (15%)	142 (14%)	816 (13%)	154 (22%)
χ^2^, *p* value	0.09, .758		110.23, <.001		23.63, <.001		0.65, .420		45.38, <.001	

Note: *p* Values based on χ^2^ test of the association between ASD (diagnosis/traits) and categorical variables, and analysis of variance (ANOVA) for differences in means for continuous variables; sample sizes vary due to differences in data availability on child, socioeconomic, parental, and familial characteristics.

Censored to prevent disclosure due to small cell counts.

**Table S2:** Distribution of Sociodemographic Characteristics in the Original Avon Longitudinal Study of Parents and Children (ALSPAC) Cohort and the Study Partial and Complete Samples

Sociodemographic Characteristics	Core ALSPAC Sample: Lacking Exposure or Outcome Data (n = 14,684)	Partial Sample: Exposure and Outcome Data Available (n = 4,479)	Complete Sample: Exposure, Outcome, and Confounders Data Available (n = 2,726)
	n (%)	n (%)	n (%)
Child characteristics			
Sex			
Male	7,536 (51)	1,787 (42)	1,171 (44)
Female	7,148 (49)	2,430 (58)	1,468 (56)
Ethnicity			
White	11,468 (95)	3,936 (96)	2,563 (97)
Nonwhite	609 (5)	144 (4)	76 (3)
Socioeconomic position			
Parental social class			
Professional/managerial	5,873 (51)	2,512 (63)	1,710 (65)
Manual	5,620 (49)	1,475 (37)	929 (35)
Accommodation type			
Detached house	1,895 (17)	896 (22)	624 (24)
Semi-detached house	7,702 (69)	2,777 (69)	1,837 (70)
Flat/apartment	1,497 (14)	343 (9)	178 (6)
Maternal educational attainment			
Degree	1,599 (14)	894 (22)	615 (23)
A-level	2,791 (24)	1,175 (29)	789 (30)
O-level	5,518 (47)	1,652 (41)	1,071 (41)
Less than O-level	1,738 (15)	298 (8)	164 (6)
Financial problems			
No financial problems	10,284 (78)	3,485 (84)	2,399 (91)
Financial problems	2,869 (22)	675 (16)	240 (9)
Early adversity			
Parental history of suicide			
No parental suicide attempt	8,033 (98)	3,917 (99)	2,607 (99)
Parental suicide attempt	146 (2)	54 (1)	32 (1)
Childhood sexual abuse			
No child sexual abuse	8,751 (99)	4,012 (99)	2,631 (99)
Childhood sexual abuse	50 (1)	19 (1)	8 (1)
Parental cruelty to children			
No	4,872 (95)	3,003 (95)	2,520 (95)
Yes	240 (5)	141 (5)	119 (5)
Maternal age and mental health			
Maternal age, mean (SD)	27.3 (5.0)	28.8 (4.4)	29.1 (4.3)
Maternal antenatal anxiety, mean (SD)	4.5 (3.3)	3.64 (3.74)	4.3 (3.2)
Maternal antenatal depression			
No	8,740 (76)	3,245 (84)	2,294 (87)
Yes	2,697 (24)	611 (16)	345 (13)
Maternal early postnatal depression			
No	9,060 (84)	3,499 (88)	2,378 (90)
Yes	1,756 (16)	470 (12)	261 (10)

**Table S3:** Associations Between Autism Spectrum Disorder (ASD vs. No ASD), Autistic Trait Measures (High vs. Low ASD Risk Group), and Self-Harm With/Without Suicidal Intent^a^ (vs. No Self-Harm) at 16 Years in Imputed Sample (n = 5,093)

Exposure/Risk Group (Age of Assessment)	Unadjusted						Adjusted^b^					
	Self-Harm Without Suicidal Intent			Self-Harm With Suicidal Intent			Self-Harm Without Suicidal Intent			Self-Harm With Suicidal Intent		
	RR	(95% CI)	*p*	RR	(95% CI)	*p*	RR	(95% CI)	*p*	RR	(95% CI)	*p*
ASD diagnosis (diagnosed by 11 y)	0.83	(0.38, 1.81)	.645	0.79	(0.29, 2.19)	.654	1.06	(0.48, 2.38)	.876	1.06	(0.37, 3.06)	.910
Reduced social communication (91 mo)	1.03	(0.71, 1.48)	.880	2.29	(1.61, 3.26)	<.001	1.17	(0.81, 1.71)	.401	2.29	(1.56, 3.36)	<.001
Repetitive behavior (69 mo)	0.86	(0.56, 1.30)	.470	1.64	(1.07, 2.50)	.022	0.92	(0.60, 1.42)	.724	1.58	(1.00, 2.48)	.049
Reduced sociability (38 mo)	0.92	(0.68, 1.24)	.569	0.92	(0.63, 1.33)	.647	0.99	(0.73, 1.34)	.946	0.99	(0.67, 1.45)	.958
Reduced coherence (115 mo)	0.80	(0.55, 1.16)	.237	1.86	(1.32, 2.62)	<.001	0.92	(0.63, 1.34)	.671	1.97	(1.36, 2.81)	<.001

Note: RR = relative risk.

a Suicidal self-harm refers to lifetime self-harm with suicidal intent; individuals in this group may also have engaged in episodes of nonsuicidal self-harm.

b Adjusted for child characteristics (sex and ethnicity), socioeconomic position (financial problems, maternal educational attainment, parental social class, and accommodation type), maternal characteristics and mental health (maternal age, antenatal anxiety, and antenatal and early postnatal depression), family history of suicide (parental suicide attempt); and early adverse experiences (childhood sexual abuse and physical cruelty to children in the household by mother/partner).

**Table S4:** Associations Between Autism Spectrum Disorder (ASD vs. No ASD), Autistic Trait Measures (High vs. Low ASD Risk Group), and Suicidal Thoughts (vs. No Suicidal Thoughts) and Plans (vs. No Suicidal Plans) at 16 Years in Imputed Sample (n = 5,093)

Exposure/Risk Group (Age of Assessment)	Suicidal Thoughts						Suicidal Plans					
	Unadjusted			Adjusted^a^			Unadjusted			Adjusted^a^		
	RR	(95% CI)	*p*	RR	(95% CI)	*p*	RR	(95% CI)	*p*	RR	(95% CI)	*p*
ASD diagnosis (diagnosed by 11 y)	0.88	(90.49, 1.57)	.664	1.06	(0.60, 1.89)	.835	0.30	(0.04, 2.14)	.232	0.37	(0.05, 2.61)	.320
Reduced social communication (91 mo)	1.47	(1.18, 1.82)	.001	1.45	(1.17, 1.79)	.001	2.00	(1.35, 2.97)	.001	1.92	(1.28, 2.88)	.002
Repetitive behavior (69 mo)	1.25	(0.94, 1.62)	.092	1.22	(0.94, 1.58)	.135	1.69	(1.06, 2.69)	.026	1.63	(1.03, 2.58)	.035
Reduced sociability (38 mo)	1.03	(0.83, 1.27)	.771	1.09	(0.88, 1.34)	.424	0.78	(0.48, 1.25)	.297	0.82	(0.51, 1.32)	.411
Reduced coherence (115 mo)	1.06	(0.84, 1.35)	.604	1.09	(0.87, 1.38)	.441	1.64	(1.09, 2.47)	.017	1.61	(1.08, 2.41)	.019

Note: RR = relative risk.

a Adjusted for child characteristics (sex and ethnicity), socioeconomic position (financial problems, maternal educational attainment, parental social class, and accommodation type), maternal characteristics and mental health (maternal age, antenatal anxiety, and antenatal and early postnatal depression), family history of suicide (parental suicide attempt); and early adverse experiences (childhood sexual abuse and physical cruelty to children in the household by mother/partner).

**Table S5:** Estimates of the Direct Effect and Effect Mediated^a^ Through Depressive Symptoms in the Association Between Impaired Social Cognition and Self-Harm at 16 Years Unadjusted and Adjusted for Antenatal Confounders in Imputed Sample (n = 7,788)

Effect Size^b^	Model Estimates					
	Unadjusted Model			Adjusted Model^c^		
	*β*	(95% CI)	*p*	*β*	(95% CI)	*p*
Total Effect Reduced social communication on self-harm	0.196	(0.04, 0.35)	.011	0.282	(0.10, 0.46)	.002
Indirect Effect Reduced social communication on self-harm, through depressive symptoms	0.094	(0.06, 0.13)	<.001	0.099	(0.06, 0.14)	<.001
Direct Effect Reduced social communication on self-harm, adjusted for depressive symptoms	0.102	(−0.04, 0.25)	.184	0.183	(0.003, 0.36)	.046

Note:

a Bias corrected (500 bootstrap samples).

b Effect size are unadjusted and adjusted regression coefficients (β unstandardized).

c Adjusted for child (sex), socioeconomic (maternal educational attainment), maternal (antenatal anxiety), and familial (parental suicidal attempt) characteristics.

## Supplemental Material

### Supplement 1

#### Method: Mediation Effects

We examined whether depressive symptoms in early adolescence may be a possible mediating mechanism in the association between childhood autism spectrum disorders (ASD) and suicidal ideation and behavior in adolescence. We hypothesized that childhood ASD would be associated with higher risk of depressive symptoms in early adolescence (12 years), and that this would constitute an indirect pathway between ASD and increased risk of self-harm with and without suicidal intent at 16 years. We used structural equation modeling to test this hypothesis, using a latent factor to encapsulate the continuum nature of depressive symptoms and by adjusting our analyses for a range of individual, maternal, and familial confounders. To improve causal inferences strongly advocated by recent methodological advancements in statistical techniques to assess mediation,^1^ we used mediation approach recommended by Muthén (unpublished manuscript, October 28, 2011; available online https://pdfs.semanticscholar.org/776d/2df784e67ab691bbffc280d3f4a480740300.pdf) to estimate mediation effects within the context of possible confounding. In line with a causal mediation framework,^2^ we assumed the following: (1) no unmeasured confounding of the exposure−outcome association; (2) no unmeasured confounding of the mediator−outcome association; (3) no unmeasured confounding of the exposure−mediator association; and (4) no mediator−outcome confounder affected by exposure. The “Model Constraint” command was used to derive new parameters and standard errors representing causally defined direct and indirect (mediated) effects.^3^ (For a detailed description and MPlus input syntax, see Muthén, unpublished manuscript, cited above). A weighted least-squares estimator was used because of its robustness in estimating both continuous and categorical variables.^4^ First, we estimated the unadjusted mediation model that included only exposure, mediator, and outcome. Second, we estimated the model adjusted for child and maternal characteristics, as well as indices of socioeconomic disadvantage. All confounders were modeled as binary indicators. Given the nature and timing of the confounders, we assumed no further intermediate confounding. Indirect effects and associated 95% confidence intervals were calculated using bias-corrected bootstrapping (500 replications) to account for the nonnormal distribution of the indirect effect.^5^

## Supplement 2

### Method: Multiple Imputation for Missing Data

We accounted for the loss to follow-up by repeating primary (n = 5,093) and mediation (n = 7,788) analyses on an imputed dataset comprising those participants with complete data on at least one exposure, one outcome, and mediator. We used multivariable imputation by chained equations (MICE)^6^ to impute missing data using the *ice* command in Stata version 13 (StataCorp, College Station, TX). The availability of rich, prospectively collected data on a range of sociodemographic variables in the Avon Longitudinal Study of Parents and Children (ALSPAC) enables one to account for confounders and factors that explain missingness, thus, supporting the missing-at-random (MAR) assumption.^7^ A total of 70 imputed datasets by 10 cycles of regression were generated. Each imputation model used all variables in the substantive analyses, along with more than 30 auxiliary variables (full list available upon request) relating to sociodemographic adversity and maternal and child psychopathology that have been identified as strong predictors of missingness in the confounders and the mediator. Monte Carlo errors were less than 10% of the standard error, and the Fraction of Missing Information values were no larger than 0.7, suggesting that 70 datasets were sufficient.^8^ The primary analysis was repeated by averaging parameter estimates over the imputed datasets and computing the associated standard error using Rubin’s rule.^9^ Standard errors and 95% confidence intervals for direct and indirect effects in the mediation analysis performed on the imputed dataset were estimated using bootstrapping (500 samples) methodology.^10^

## Supplement 3

### Results: Missing Data: Sensitivity Analyses

The results from analyses with imputed data supported our substantive findings, providing further evidence to support the association between impaired social communication skills in early childhood and risk of self-harm with suicidal intent, suicidal thoughts, and plans in late adolescence. In contrast to the complete case analysis, there was also evidence for the main effect of repetitive behavior and reduced speech coherence on risk of self-harm with suicidal intent and suicidal plans (Tables S3 and S4). In the mediation models, the direct and indirect effect estimates were in the same direction and led to the same overarching conclusions (Table S5). However, the sizes of the observed direct and indirect effects were greater in the analysis with the imputed data. The pattern of missing data and analyses suggested that attrition led to a possible underestimation of the direct and indirect effect sizes in the complete case analyses. In particular, within the fully adjusted model using imputed data, the indirect path from impaired social communication to self-harm through depressive symptoms was estimated as product coefficient [β] = 0.099, 95% CI = 0.06−0.14, *p* < .001, whereas the total effect was estimated as regression coefficient [β] = 0.282, 95% CI = 0.10−0.46, *p* = .002. Based on analyses using imputed data, we would estimate that a slightly higher proportion (35%) of the total association between impaired social communication and self-harm is accounted for by the indirect path through depressive symptoms.

### Association Between ASD and Confounding Variables

Overall, compared to adolescents without ASD, adolescents with ASD diagnosis and autistic traits were more likely to be male, to come from less socioeconomically advantaged families (as indexed by lower parental social class, worse housing, lower levels of maternal education, and presence of financial problems), and had older mothers who reported higher levels of perinatal mental health problems (anxiety and depression; Table S1).
